# Celiac Disease, Enteropathy-Associated T-Cell Lymphoma, and Primary Sclerosing Cholangitis in One Patient: A Very Rare Association and Review of the Literature

**DOI:** 10.1155/2013/838941

**Published:** 2013-12-05

**Authors:** N. Majid, Z. Bernoussi, H. Mrabti, H. Errihani

**Affiliations:** ^1^Department of Medical Oncology, National Institute of Oncology, Rabat 10100, Morocco; ^2^Department of Pathology, University Hospital of Avicenne, Rabat, Morocco

## Abstract

Enteropathy-associated T-cell lymphoma (EATL) is a very rare peripheral T-cell lymphoma which is mostly associated with celiac disease. However, the association of primary sclerosing cholangitis and enteropathy-associated T-cell lymphoma is uncommon. Herein we report and discuss the first case of patient who presented simultaneously with these two rare diseases. It is a 54-year-old man who stopped gluten-free diet after 15 years history of celiac disease. The diagnosis was based on the histological examination of duodenal biopsy and the diagnosis of primary sclerosing cholangitis was made on liver biopsy, as well as the magnetic resonance cholangiogram. The treatment of EATL is mainly based on chemotherapy in addition to the optimal management of complications and adverse events that impact on the response to treatment and clinical outcomes, although the prognosis remains remarkably very poor.

## 1. Introduction

Celiac disease is a common systemic disorder. It is associated with autoimmune disorders and increased risk of malignancy including an increased risk of non-Hodgkin's lymphoma especially of the T-cell type [1].

Enteropathy-associated T-cell lymphoma (EATL) is a rare form of aggressive-cell lymphoma which accounts for fewer than 5% of all gastrointestinal tract lymphomas [2]. It is classified into two groups based on morphology, immunohistochemistry, and genetic profile: type I is more common in frequency and highly associated with celiac disease compared with type II [3]. Concerning primary sclerosing cholangitis (PSC) it is defined as a chronic cholestatic liver disease of unknown aetiology characterised by inflammation and fibrosis of the biliary tree [4]. The relationship between PSC and celiac disease remains unknown, although an immunologic connection is suspected.

The outcome is very poor with an overall survival rate of 15–20% in 2 years [5]. Thus, the objective of this paper is to remind clinicians about the importance of adherence to a gluten-free diet that decreases the risk of developing enteropathy-type T-cell lymphoma with its associated autoimmune diseases and poor prognosis.

## 2. Case Presentation

Herein we describe a case of a 54-year-old man who stopped gluten-free diet for more than 8 years after a 15-year history of celiac disease (antiendomyseal and antitransglutaminase antibody tests were positive). The patient was admitted to the hospital for evaluation of vomiting and weight loss; the history was negative for fever or night sweats. Clinical examination revealed pallor of the skin, splenomegaly +1 cm without ascites or lymphadenopathy. The heart and lungs examination was unremarkable.

**Figure 1:**
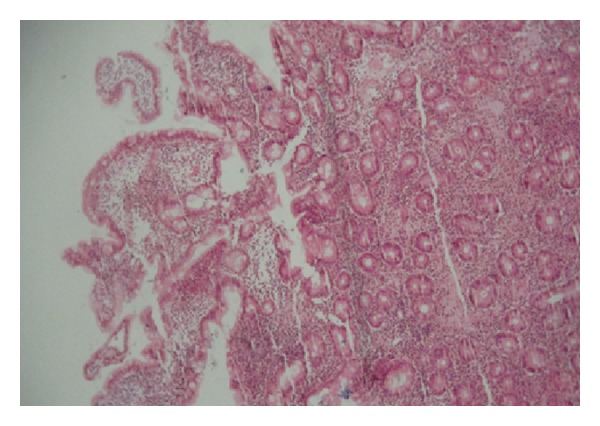
Histologic examination showing a moderate lymphocyte infiltration expanding villus axis and lamina propria. H&E stain (×200).

**Figure 2:**
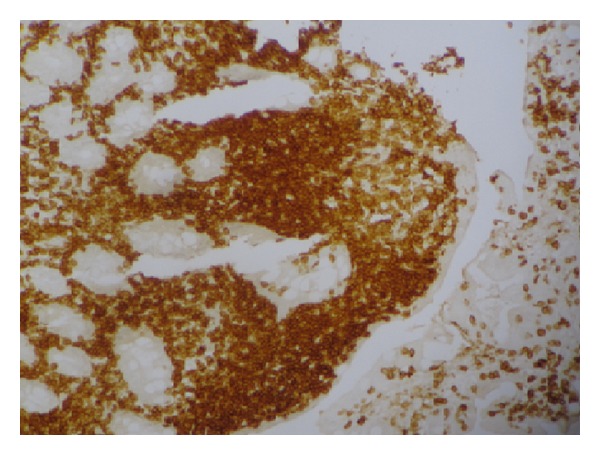
Immunohistochemical study showing diffuse positivity for CD 30 (×200).

Hematological investigations revealed a hemoglobin of 10 g/dL and LDH of 251 IU/L. Liver function test results were within normal ranges except for GGT = 87 IU/L (9–64) and alkaline phosphatase (ALP) = 228 IU/L (40–150). Serological tests for HIV and hepatitis (B and C) were negative. Anatomopathological and immunohistochemical analysis of duodenal biopsy after esophagogastroduodenoscopy revealed total to subtotal villous atrophy with increased intraepithelial lymphocytes (IELs), associated with numerous inflammatory cells, and diffuse polymorphic lymphoid cells strongly positive for CD3, but negative for CD20, CD8, and CD56 consistent with enteropathy-associated T-cell lymphoma (Figures 1 and 2). PCR analysis showed monoclonal rearrangement of the TCR*γ* chain. Abdominal ultrasound revealed signs of portal hypertension without causes of biliary obstruction. The diagnosis of primary sclerosing cholangitis was confirmed by the results of magnetic resonance cholangiogram that shows moderate stricturing of intrahepatic bile ducts alternating with areas of dilatation. Additionally, liver biopsy shows mild portal inflammation with infiltration of lymphocytes in the bile duct and ductular proliferation. Staging computerized tomography of her chest and abdomen was performed and revealed the presence of confluent lymph nodes in the mesenteric and paraaortic areas. Thus the patient was diagnosed as stage II_E_ according to the Ann Arbor staging system. After 4 cycles of chemotherapy (CHOP regimen), one episode of febrile neutropenia occurred and the disease was stable. Then the decision was to start second line chemotherapy with ICE regimen (ifosfamide, carboplatin, and etoposide) followed by autologous stem cell transplantation. The patient has just received the first course of this protocol.

## 3. Discussion

Enteropathy-associated T-cell lymphoma (EATL) is a relatively rare peripheral T-cell lymphoma with the incidence rate less than 1% of all NHL and fewer than 5% of all gastrointestinal tract lymphomas [2]. EATL can be divided into two groups: type I is highly associated with celiac disease and predominates in Western countries, while type II is not associated with celiac disease and is more common in Asia [3]. The median age at diagnosis is 60 years in patients presenting the classic symptoms of celiac disease, such as abdominal pain, diarrhea, and weight loss together with fever and night sweating. The clinical symptoms may also include abdominal emergencies such as spontaneous intestinal perforation, obstruction, or hemorrhage.

A high level of clinical suspicion should lead to staging procedures as recommended for lymphoma including thoracic and abdominal imaging, endoscopy, and histologic examination of intestinal and bone marrow biopsies. The histologic examination of EATL type I shows medium-sized to large tumor cells with an increased mitotic index associated with histologic features of active celiac disease (CD), such as crypt hyperplasia, increased IEL infiltration, and villous atrophy. Immunophenotype is typically CD3+, CD5–, CD7+, CD8+/–, CD4–, CD30+ [6]. Moreover, the PCR analysis is indicative for a monoclonal rearrangement of the TCR*γ*-chain on intestinal tissue sections, and a flow cytometric characterization of IELs shows an aberrant T-cell population that predicts the progression of recurrent celiac disease (RCD) into EATL [7].

Giving this similarity with the profile of our case, we can conclude to the diagnosis of EATL type I in our patient with the peculiarity of having duodenal location considered rare comparatively to small bowel anatomic site.

Primary sclerosing cholangitis (PSC) is a chronic cholestatic liver disease of unknown etiology, characterized by inflammation and fibrosis of the biliary tree [8]. It is normally associated with inflammatory bowel disease especially ulcerative colitis, but less frequently with other autoimmune diseases such as celiac disease, diabetes mellitus, and rheumatoid arthritis. The etiology and pathogenesis of this disease are still unknown but an immune mediated basis is commonly admitted [9]. The combination of non-Hodgkin lymphoma and PSC is extremely uncommon; only rare cases were reported in an extensive search in the medical literature, but to our knowledge none had simultaneous occurrence of PSC and EATL as presented in this case. We suggest that this intriguing association may possibly be mediated by immunogenetic factors.

PSC is a progressive disease with unknown etiology, where liver transplant remains the only effective therapeutic option for patients with end-stage liver disease [10].

Concerning the management of EATL in patients with CD, there is no validated standard but the therapy is the same as for patients without celiac disease.

Surgery is especially reserved to the treatment of gastrointestinal complications that can occur during chemotherapy or reveal the diagnosis [11]. However, combination chemotherapy is the treatment most frequently used in clinical practice, the majority of patients cannot receive or complete the treatment because of poor performance status, complications, and their intrinsic frailty. Interestingly, the CHOP regimen (cyclophosphamide, doxorubicin, vincristine, and prednisone) is the most widely used with an overall 5-year survival of 9% to 22% [12]. High-dose chemotherapy followed by autologous stem cell transplantation (ASCT) was also tested retrospectively and has yielded to durable disease control in a significant proportion of patients who were very strictly selected [13].

Our patient was also proposed for second line chemotherapy followed by ASCT because of his good performance status and liver function tests. The challenge is the optimal management of complications and adverse events that impact on the response to treatment and thus improved clinical outcomes, although the prognosis remains poor and the 2-year survival rate is of 15 to 20% [5].

## 4. Conclusion

This is the first case in the published literature, which describes the simultaneous occurrence of two rare diseases: PSC and EATL. The aim of this paper is to emphasize the importance of strict adherence to a gluten-free diet, as it can decrease the risk of developing enteropathy-associated T-cell lymphoma and possibly associated autoimmune diseases. Additionally, patients who are unresponsive to a gluten-free diet or with deteriorating clinical condition should be investigated for the development of lymphoma.
